# Is Laterality Prognostic in Resected KRAS-Mutated Colorectal Liver Metastases? A Systematic Review and Meta-Analysis

**DOI:** 10.3390/cancers14030799

**Published:** 2022-02-04

**Authors:** Michail Belias, Kazunari Sasaki, Jane Wang, Nikolaos Andreatos, Carsten Kamphues, Georgios Kyriakos, Hendrik Seeliger, Katharina Beyer, Martin E. Kreis, Georgios Antonios Margonis

**Affiliations:** 1 Health Evidence, Radboud University Medical Center, Mailbox 133, P.O. Box 9101, 6500 HB Nijmegen, The Netherlands; michaelmpelias@gmail.com; 2 Department of General Surgery, Digestive Disease Institute, Cleveland Clinic, Cleveland, OH 44195, USA; sasakik@ccf.org (K.S.); andreatos.nikolaos@mayo.edu (N.A.); 3 Department of Surgery, University of California San Francisco, San Francisco, CA 94143, USA; jaeyunjane.wang@ucsf.edu; 4 Department of General and Visceral Surgery, Charité Campus Benjamin Franklin, 12200 Berlin, Germany; carsten.kamphues@charite.de (C.K.); hendrik.seeliger@iu.org (H.S.); katharina.beyer2@charite.de (K.B.); martin.kreis@charite.de (M.E.K.); 5 Division of Endocrinology and Nutrition, Hospital General Universitario Santa Lucia, 30202 Cartagena, Spain; gkyriakos1@alumno.uned.es; 6 Department of Surgery, Memorial Sloan Kettering Cancer Center, New York, NY 10065, USA

**Keywords:** primary tumor location, KRAS mutated, KRAS wild type, colorectal liver metastases, laterality

## Abstract

**Simple Summary:**

Primary tumor laterality (PTL) is the most recently identified prognostic factor associated with mortality in patients with resected colorectal cancer liver metastases, but whether it is prognostic in all patients or only those with wild-type KRAS tumors is debated. The aim of this meta-analysis was to identify all relevant articles and synthesize their evidence to estimate the effect of PTL per KRAS mutational status. We found that PTL and KRAS mutational status have a statistically significant interaction. Specifically, PTL has a variable effect in patients with wild-type versus KRAS-mutated tumors, with right-sided tumors associated with worse survival only in the former. This meta-analysis appears to resolve a long-lasting debate.

**Abstract:**

Background: It is debated whether primary tumor laterality (PTL) is prognostic in all patients with colorectal liver metastases (CRLM) or only those with KRAS wild-type or KRAS-mutated tumors; Methods: We systematically reviewed PubMed for studies reporting on resected CRLM originating from left-sided (LS) versus right-sided (RS) colon cancer stratified by KRAS status. Individual participant data (IPD) were used if available. Given that there are two definitions of PTL, we performed two meta-analyses for KRAS-mutated and two for wild-type patients. To assess if an interaction underlies the possible difference between the effects of PTL in KRAS-mutated vs. wild-type CRLM, we similarly performed two meta-analyses of interaction terms; Results: The meta-analyses included eight studies and 7475 patients. PTL had a prognostic association with OS in patients with wild-type tumors (HR for LS: 0.71 [0.60–0.84]), but not in those with KRAS-mutated tumors (HR: 0.99 [0.82–1.19]). This difference stemmed from a truly variable effect of PTL for each KRAS status (mutated vs. wild-type) as the meta-analysis of interaction terms showed a significant interaction between them (HR:1.38 [1.24–1.53]). Similar results were obtained when the second definition of PTL (LS to not include the rectum) was used; Conclusions: KRAS status modifies the association of tumor site with survival. Right-sided tumors are associated with worse OS only in patients with wild-type CRLM.

## 1. Introduction

Primary tumor laterality (PTL) is the most recently identified prognostic factor associated with mortality in colon cancer, particularly in the metastatic setting (metastatic colorectal cancer (mCRC)). The molecular underpinnings of the prognostic difference between right-sided (RS) vs. left-sided (LS) tumors include the higher rates of deleterious somatic mutations (e.g., BRAF, KRAS, and SMAD4) in RS tumors [1]. A notable exception is TP53, which is more commonly found in LS tumors [1]. Another possible exception is microsatellite instable (MSI) tumors, which are more likely to be found in the right colon and are associated with better prognosis in nonmetastatic colon cancer. Of note, PTL also has a predictive role in unresectable metastatic colon cancer and can predict treatment benefit from targeted therapy [2,3].

In 2016, Sasaki et al. suggested that PTL may be associated with worse overall survival (OS) in patients with resected colorectal cancer liver metastases (CRLM) [4]. Since then, most studies in patients with CRLM confirmed that RS primary tumors may show worse OS [5], although others did not show a relationship between PTL and long-term mortality [6,7]. Wang et al. performed the first meta-analysis which showed that RS tumors have worse OS than LS tumors [8]. However, their meta-analysis showed high heterogeneity, implying that a subgroup effect may be present. 

In 2019, another group suggested that this subgrouping variable may be the KRAS mutational status [9]. Specifically, they showed that patients with RS tumors had worse OS than those with LS tumors, but only in patients with wild-type KRAS status and not in those with KRAS mutations [10]. However, other groups have published contradictory findings.

To offer a potential resolution to this debate, we performed a systematic literature search and meta-analysis using all relevant studies. Given that LS disease can be defined in two possible ways (excluding or including rectal tumors), we performed two sets of meta-analyses using each definition. Each set included separate meta-analyses for KRAS-mutated and wild-type patients as well as for the interaction between tumor site and KRAS status.

## 2. Materials and Methods

### 2.1. Objective

The present study aimed to determine whether the effect of PTL on OS was different between patients with KRAS-mutated and KRAS wild-type CRLM who underwent metastasectomy. The reporting of this systematic review followed the Preferred Reporting Items for Systematic Review and Meta-Analyses (PRISMA) statement (eFile S1 in the supplement) [11]. 

### 2.2. Data Sources and Search Strategies

We performed a comprehensive literature search in the PubMed database for full-text articles published in print or online from inception until May 2021 (the protocol was not registered). The detailed search strategy is described in eFile S2 in the supplement. The search strategy was designed and conducted by an experienced librarian (A.T.) with input from the study investigators. Two authors (M.B and G.A.M.) independently identified and reviewed full-text articles that were deemed relevant by screening their titles and abstracts. Disagreements between the two reviewers were resolved with consensus. We also manually included relevant studies using the similar articles function of Pubmed. 

### 2.3. Inclusion Criteria

We included original studies that either reported the effect of PTL stratified by KRAS status as a hazard ratio (or any other relevant effect size) or showed Kaplan–Meier plots stratified by KRAS status. The outcome of interest was 5-year OS measured from the date of initial CRLM surgery. We excluded studies not written in English, Dutch, Greek, Spanish, or German. When we encountered more than one study published by the same authors, we selected the newest or most informative article.

### 2.4. Data Extraction

For eligible studies authored by the senior author (G.A.M) or his collaborators from the International Genetic Consortium for Colorectal Liver Metastasis (IGCLM), we received and used individual participant data (IPD). As noted in the Cochrane handbook for systematic reviews of interventions, the IPD approach can bring substantial improvements to the quality of data available and can offset inadequate reporting of individual studies [12]. For the remaining studies, we used aggregate data (AD) or simulated IPD using the Kaplan–Meier plots. 

One author (M.B.) extracted prespecified data elements from the eligible studies, including study-specific information and the outcome of interest. Study-specific information included author name, year of publication, country, study interval, number of patients, definition of right vs. left side (and whether rectum was included in LS), location of the primary tumor, and KRAS mutational status. 

The outcome of interest was Hazard Ratio (HR) for OS. If other relevant effect size indices were used, we transformed them to HR. If the survival information was only presented in Kaplan–Meier survival curves, we simulated their IPD based on the method developed by Guyot et al. [13].

### 2.5. Statistical Analysis 

To estimate the effect of PTL per KRAS mutational status, we performed two separate sets of meta-analyses according to the two different definitions of PTL. For each study where IPD were available, we first applied an univariable Cox Proportional Hazard (PH) model per KRAS mutational status group. We extracted their HR along with their standard errors. Subsequently, we combined them along with the corresponding AD estimates using both a fixed and random effect meta-analysis with empirical Bayes τ^2^.

To assess the difference between the effects of PTL across the KRAS subgroups, we performed a meta-analysis of interaction terms. For each study, we applied a Cox PH model including KRAS, PTL, and their interaction term (KRAS × PTL). Subsequently, we pooled the extracted estimates from IPD with the corresponding AD using both a fixed and random-effects meta-analysis with empirical Bayes τ^2^ [14]. Given that recent studies have shown that the DerSimonian and Laird approach for a random-effects meta-analysis may be suboptimal, we instead used the method described by Hartung and Knapp and by Sidik and Jonkman (i.e., the HKSJ method) [15]. To assess for study heterogeneity, we used the I^2^ statistic. In cases of high heterogeneity, we reported the random-effects meta-analysis pooled estimate and showed both fixed and random-effects pooled estimates in their forest plots. By convention, an observed HR of <1 implies better survival for patients with LS cancers. Two-sided *p* < 0.05 was deemed statistically significant. To inform clinicians of what effect to expect in future studies, we also reported the 95% prediction intervals of the pooled estimates along with the 95% CIs [16].

#### 2.5.1. Sensitivity Analysis

To assess the consistency of our results and test whether potential confounding is present, we applied a multivariable mixed-effects Cox–PH model. Similar to the above analysis, we included KRAS, PTL, and their interaction term (KRAS × PTL) along with gender, age at the time of surgery, lymph node status, receipt of pre-hepatectomy chemotherapy, presence of resected extrahepatic disease, use of concurrent intraoperative ablation, and synchronous or metachronous presentation of the liver metastases. To account for within study clustering of the participants, we assumed random slope for KRAS, PTL, and their interaction term (KRAS × PTL) and fixed slope for remaining variables. To avoid overfitting, we used Ridge penalty for the latter. As in the meta-analysis described above, the estimate of interest was the interaction term (KRAS × PTL). We considered the estimate of the multivariable model as consistent if within the 95% confidence interval of the pooled random-effects estimate of the meta-analysis. If a reverse effect was shown, we considered the estimate of the multivariable model as inconsistent. Otherwise, the result was deemed inconclusive. 

#### 2.5.2. Bias Assessment

We did not perform a risk of bias assessment as the quality of the studies was expected to be similar (all were retrospective studies of observational data). 

#### 2.5.3. Publication Bias

To assess whether publication bias was present, we performed both a rank correlation and linear regression test for funnel asymmetry [17].

### 2.6. Statistical Packages

All analyses were performed using the statistical software R version 3.6.0 (26 April 2019). We used the tidyverse package for data manipulation, the survival package for the Cox PH, and the meta package for the meta-analysis. 

## 3. Results

### 3.1. Study Selection

**Figure 1:**
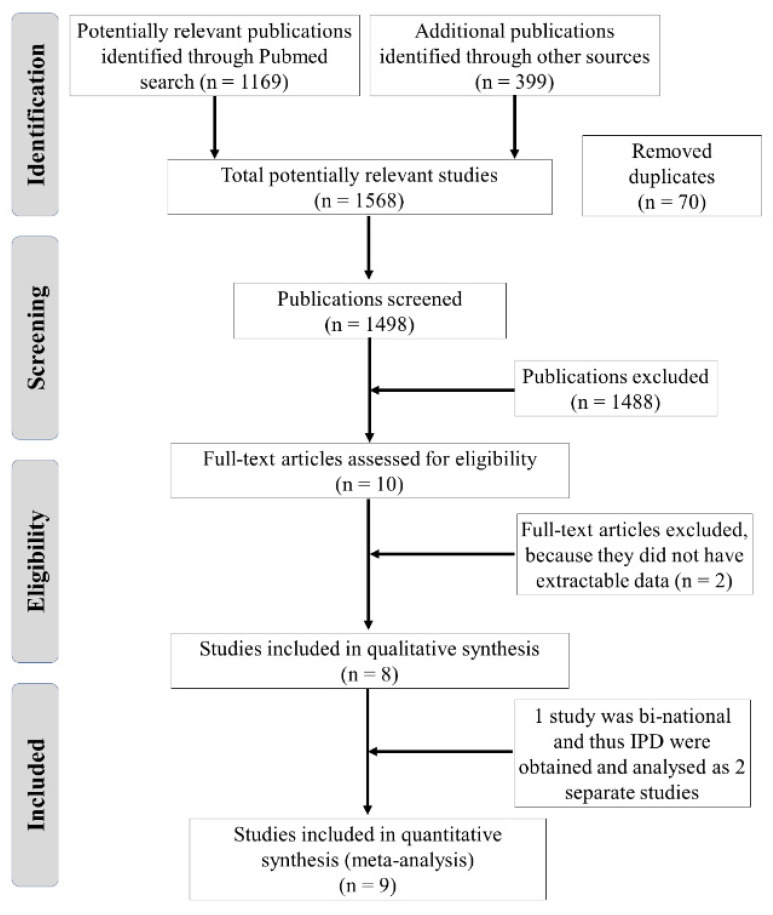
Overview of the study search and selection.

A total of 1169 titles and abstracts were identified by the search strategy. After title and abstract screening, 10 articles met the eligibility criteria (PRISMA chart, Figure 1). After full text inspection, eight articles had extractable data and were included in the meta-analysis. IPD data were obtained for three studies, although the study by Gagniere et al. was binational and thus IPD were obtained and analyzed separately (as “Gagniere et al.” for the MSKCC dataset and “French datasets” for the University of Lyon and Clermont-Ferrand University datasets). AD data were used for the other four studies. 

### 3.2. Study Characteristics

The eight studies comprised 7475 patients ranging from 227 to 2655 patients per study (median: 645, IQR: 587.5). The major characteristics are shown in Table 1 and Table 2. With regard to the variables of interest, 1281 patients had KRAS-mutated RS tumors, 1443 had KRAS-mutated LS tumors, 1394 patients had wild-type RS tumors, and 3357 had wild-type LS tumors.

### 3.3. Meta-Analysis of Overall Survival Stratified by KRAS Mutational Status Using the First Definition of PTL

**Figure 2:**
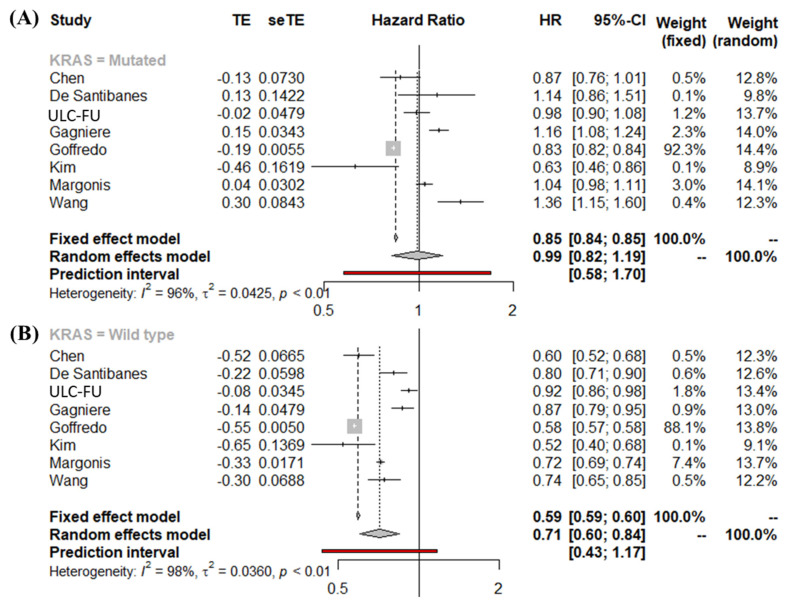
(**A**) Forest plot for right vs left (with rectum) in patients with KRAS-mutated tumors, (**B**) Forest plot for right vs left (with rectum) in patients with KRAS wild-type tumors; reference: right side.

All our meta-analyses showed high heterogeneity; therefore, we only reported the random-effects pooled HR and included the fixed-effects pooled estimate in our forest plots. For the KRAS-mutated tumors, the pooled HR was 0.99 (95% CI, 0.82–1.19) (Figure 2A), while for the KRAS wild-type tumors the pooled HR was 0.71 (95% CI, 0.60–0.84) (Figure 2B), indicating that PTL has a prognostic value only in patients with wild-type tumors. 

### 3.4. Meta-Analysis of Overall Survival Stratified by KRAS Mutational Status Using the Second Definition of PTL

**Figure 3:**
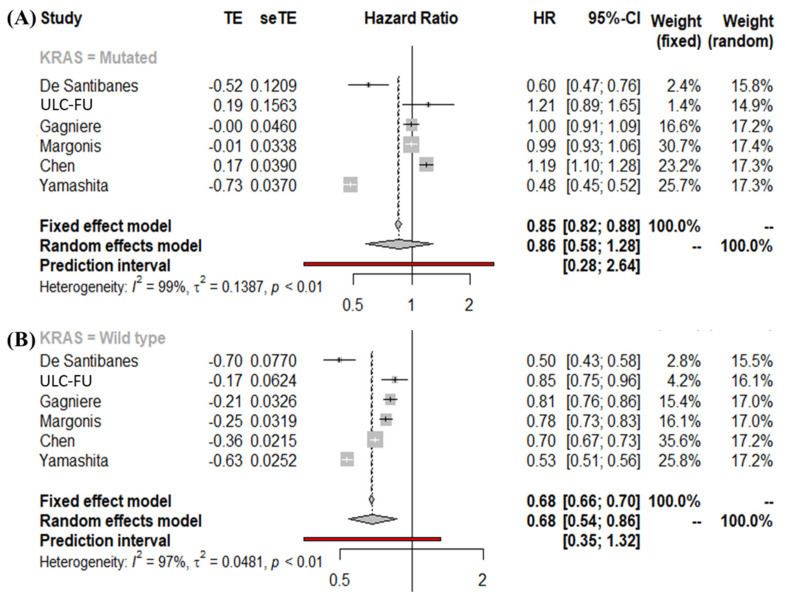
(**A**) Forest plot for right vs left (without rectum) in patients with KRAS-mutated tumors, (**B**) Forest plot for right vs left (without rectum) in patients with KRAS wild-type tumors; reference: right side.

When an alternate definition of PTL was used (patients with rectal tumors were not included in the LS group), the analysis showed similar results confirming that PTL has prognostic value only in patients with wild-type tumors. Specifically, the pooled HRs were 0.86 (95% CI, 0.58–1.28) (Figure 3A) and 0.68 (95% CI, 0.54–0.86) (Figure 3B) for KRAS-mutated and wild-type tumors, respectively. 

### 3.5. Meta-Analysis of Overall Survival Interaction Terms (MA-IT) Using the First Definition of PTL

**Figure 4:**
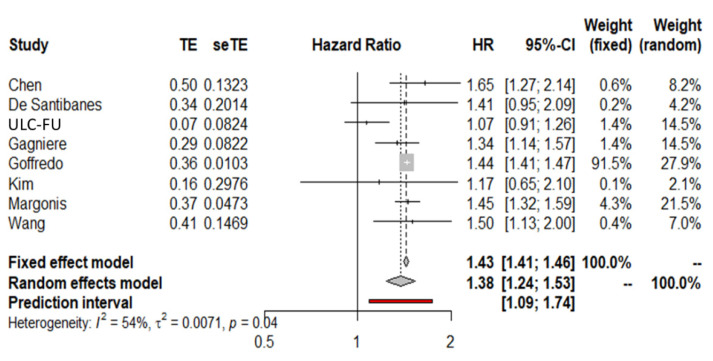
Forest plot for KRAS sidedness interaction terms (left includes rectal tumors).

The meta-analysis of interaction terms showed that there was a significant interaction between tumor side and KRAS mutational status. Specifically, the pooled HR for interaction terms was 1.38 (95% CI 1.24–1.53) (Figure 4). This indicates that there was a statistically significant difference on the effect of PTL between KRAS wild-type and KRAS-mutated tumors. The interaction term estimate in the multivariable mixed effects model was 1.35 (95% CI 0.78–2.31) and therefore consistent with the pooled HR shown above.

### 3.6. Meta-Analysis of Overall Survival Interaction Terms (MA-IT) Using the Second Definition of PTL

**Figure 5:**
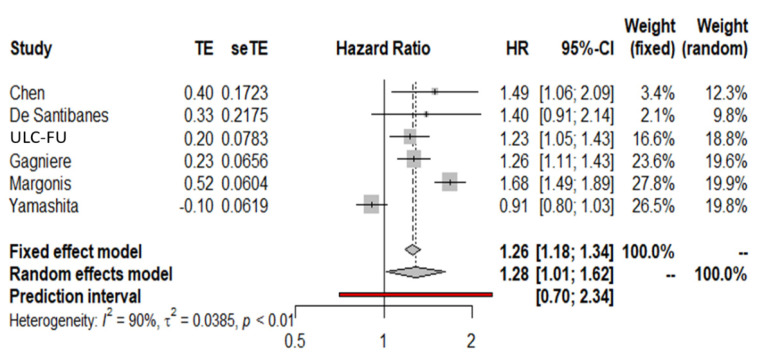
Forest plot for KRAS sidedness interaction terms (left does not include rectal tumors).

When an alternate definition of PTL was used (patients with rectal tumors were not included in the LS group), a similarly significant interaction between tumor side and KRAS mutational status was observed. Specifically, the pooled HR for interaction terms was 1.28 (95% CI 1.01–1.62) (Figure 5). The interaction term estimate in the multivariable mixed-effects model was 1.10 (95% CI 0.69–1.75) and therefore consistent with the pooled HR shown above.

Publication bias: The publication bias tests could not be performed because a minimum of 10 studies was required. 

## 4. Discussion

This meta-analysis included a large number of patients (*n* = 7475) with data on primary tumor side and KRAS mutational status. The robustness of the study was further increased by the inclusion of patient-level data (IPD). To our knowledge, it is the first meta-analysis in CRLM that investigated whether the effect of PTL is independent of or contingent on KRAS status. The study ultimately showed that PTL and KRAS mutational status have a statistically significant interaction. Specifically, PTL has a different effect in patients with wild-type versus KRAS-mutated tumors, with RS tumors associated with worse OS only in the former. The variable effect of KRAS status on PTL persisted regardless of whether patients with rectal tumors were included in the LS group. This is important because there is evidence that grouping together patients with left-sided and rectal tumors may not be methodologically and biologically sound [1,23]. 

Furthermore, our findings may explain why previous studies on PTL generated conflicting results. For example, a recent meta-analysis on PTL reported that although RS was overall associated with worse OS, about half of the included studies (22/43) did not show significant associations between RS tumors and worse OS [8]. Given that the frequency of KRAS mutations varies widely (15–38% according to a recent meta-analysis), it is possible that in smaller studies, a relatively high frequency of KRAS mutations can tip the scale in favor of no survival difference between RS and LS tumors [24]. 

Although this is the first meta-analysis to show a variable effect of PTL based on KRAS mutational status in surgically treated patients, a meta-analysis in medically treated patients with metastatic, unresectable CRC showed that the prognostic value of PTL is restricted to the KRAS wild-type population [25]. Specifically, a group from Mayo clinic analyzed data from 9277 mCRC patients from 12 first-line randomized trials and reported a statistically significant interaction between PTS and KRAS mutation. Specifically, LS disease was associated with better OS among KRAS wild-type (OS HR = 0.59, 95% CI = 0.53–0.66) but not among KRAS-mutated tumors. This is similar to the HR reported in this meta-analysis (OS HR = 0.71, 95%CI = 0.60–0.84).

Similarly, our results are consistent with those reported by Cavallaro et al., who investigated the relationship between PTL and KRAS status in a mixed National Cancer Data Base (NCDB) cohort of resectable and unresectable patients with CRC and synchronous metastases to the liver [26]. Specifically, they found that among those with wild-type tumors, the OS of patients with LS tumors was numerically superior to those with RS disease (median OS: 31.5 vs. 16.7 months, respectively), while in patients with KRAS-mutated tumors, OS was comparable (median OS of 21.1 months for those with RS tumors and 23.7 for those with LS tumors). Importantly, our findings may apply even to patients with nonmetastatic CRC. Specifically, a study by Kamphues et al. evaluated the interplay between KRAS status and PTL in a cohort of patients with nonmetastatic CRC treated at six academic centers in Europe and Japan [23]. In this cohort, KRAS mutation status was only found to be prognostic among patients with LS disease, which is consistent with the present study. 

Given the findings of this meta-analysis, it is tempting to speculate on the molecular profiles that make KRAS-mutated tumors largely indifferent to PTL. Some studies have suggested that KRAS mutation is only prognostic when there is a coexisting TP53 or SMAD4 mutation [27,28]. Interestingly, the relatively equal distribution of these two “activating” mutations between RS and LS disease may at least partially account for the similar prognosis of KRAS-mutated tumors regardless of PTL. Among patients with wild-type tumors, PTL likely impacts outcomes through other activating mutations such as BRAF V600E, which is not only largely mutually exclusive of KRAS and associated with poor prognosis but is also encountered far more frequently in RS disease. 

This meta-analysis has some inherent weaknesses that must be acknowledged. All studies included in the meta-analysis were retrospective and thus have inherent limitations including the notable heterogeneity that we observed. The lack of eligible prospective studies in patients with surgically treated CRLM stems from a general lack of prospective studies in this group due to either ethical concerns or the lack of definitive evidence that perioperative chemotherapy benefits these patients. Specifically, the inconclusive EPOC trial has discouraged further prospective studies on the impact of various perioperative systemic therapies [29]. KRAS mutational status was determined on either the resected CRLM or the primary tumor, with the caveat that primary colorectal tumors may differ from their metastases with regard to the mutational status. However, previous studies have demonstrated a high concordance in somatic gene mutational status [30]. Another limitation of the meta-analysis is that we did not include data on other somatic mutations. For example, it is possible that some tumors were misclassified as wild type if they were not tested for all relevant KRAS exon- and codon-specific mutations or if they were not tested for the BRAF mutation. Finally, a meta-regression analysis was not performed because the aim of the study was to assess whether PTL had a different effect on OS in patients with wild-type versus KRAS-mutated tumors.

## 5. Conclusions

The findings of this meta-analysis suggest that patients with RS or LS KRAS-mutated tumors have similarly poor prognoses, while those with RS wild-type tumors have a 29% increased risk of death compared to their LS counterparts. This variable effect of PTL based on KRAS status may help resolve the current debate regarding the effect of PTL on survival, which is evidenced by the fact that 21/43 published CRLM studies on PTL showed a significant association between PTL and prognosis while the other 22 did not [8]. A recent editorial posed the question of whether these results stemmed from true interactions between PTL and KRAS status or from a simple superimposition of distinct effects [31]. This meta-analysis was able to answer this question by detecting significant interaction between PTL and KRAS status. This is significant because previous studies, which demonstrated that tumor site was only significant in wild-type patients, could not prove a true variable effect of KRAS status on PTL. Taken together with the study by our group on nonmetastatic CRC and the meta-analysis published by Yin et al. on unresectable metastatic CRC, these findings suggest that the interactions between KRAS and PTL exist across multiple stages of disease, ranging from nonmetastatic CRC to resectable and unresectable metastatic disease [25]. These results have potential clinical implications. For example, a recent analysis by our group (unpublished data) showed a statistically significant interaction between KRAS and margin status. Furthermore, PTL may partially underlie the variable effect of surgical margin in KRAS-mutated tumors, as the only significant difference in patients with R1 resections and KRAS-mutated vs. wild-type tumors was the high prevalence of RS tumors in the former. Thus, KRAS, margin status and potentially PTL may be used to guide the surgical management of patients with CRLM; future studies are needed to elucidate whether PTL can predict treatment benefit in CRLM. Moreover, an integration of KRAS, PTL, margin status, and systemic treatments to optimize long-term outcomes can be achieved with potent, explainable algorithms such as optimal policy trees [32].

## Figures and Tables

**Table 1:** Study characteristics and variable of interest.

Study Characteristics	Yamashita et al. [10]	Wang et al. [7]	Goffredo et al. [18]	Gagniere et al. [19]	Margonis et al. [9]	Kim et al. [20]	Chen et al. [21]	De Santibanes et al. [22]	ULC—FU *
Publication year	2018	2018	2018	2018	2019	2020	2020	2019	2018
Country	USA	China	USA	USA	USA	South Korea	Taiwan	Argentina	France
Study type	Single	Single	Population	Multicenter	Multicenter	Single	Single	Population	Single
Retrospective Design	Yes	Yes	Yes	Yes	Yes	Yes	Yes	Yes	Yes
Interval of data collection	Nov. 1990–Feb. 2015	Jan. 2002–Dec. 2015	Jan. 2010–Dec. 2015	Jan. 2001–Dec. 2016	Jan. 2000–Dec. 2016	Jan. 2006–Dec. 2015	NR	NR	Jan. 2001–Dec. 2016
Total patients	725	420 ^a^	2665	775	1137 ^b^	227	336 ^c^	595 ^d^	695
Median FU (months)	27	26	NR	53.1	26.13	43.4	39.5	16.73	46.13
KRAS mutated ^†^	262	97	1116	299	297	78	124	147	304
Right/Left	92/170	29/68	651/465	112/187	124/173	21/57	41/83	31/116	180/124
KRAS wild type	463	233	1539	476	840	149	212	448	391
Right/Left	146/317	38/195	541/998	99/377	200/640	20/129	28/184	82/366	240/151

^a^ The study included 420 patients but only 330 of them had known KRAS status and were included in the present meta-analysis. ^b^ The study included 718 patients but IPD were available for 1137 patients (as patients with rectal tumors were excluded from the original publication) who were included in the present meta-analysis. ^c^ The study included 381 patients but only 336 of them had known KRAS status and were included in the present meta-analysis. ^d^ The study included 662 patients but IPD were available for 595 of them who were included in the present meta-analysis. NR: not reported. * University of Lyon and Clermont-Ferrand University. ^†^ Somatic Gene Mutation Profiling: Yamashita et al.: tested for KRAS codons 12 and 13 in all patients and KRAS codons 61 and 146 and NRAS codons 12, 13, and 61 in the majority of patients; Wang et al.: tested for KRAS codons 12, 13 and 61 and NRAS codons 12, 13 and 61; Goffredo et al.: tested for KRAS status of the primary tumor; Gagniere et al.: NR; Margonis et al.: tested for KRAS codons 12, 13, and 61, with the exception of the patients from Haukelund University, who only underwent sequencing of codons 12 and 13. Patients from the early 2000s were not tested for all mutations in exons 3, and none of the patients were tested for exon 4 mutations; Kim et al.: tested for KRAS codons 12 and 13; Chen et al.: tested for KRAS; NRAS mutation was not tested; De Santibanes et al.: tested for exon 2 (codons 12 and 13), exon 3 (codon 61) and exon 4 (codons 117 and 146) KRAS mutations; ULC-FU: NR.

**Table 2:** Demographic-, tumor- and treatment-related patient characteristics.

Patient Characteristics	Yamashita et al. [10]	Wang et al. [7]	Goffredo et al. [18]	Gagniere et al. [19]	Margonis et al. [9]	Kim et al. [20]	Chen et al. [21]	O Connoret al. [22]	ULC—FU *
**Age, median [interquartile range]/Age, mean ± SD**	58 (50–65)	NR	NR	NA	61 (51–68)	NR	NR	67 (59–75)	59.6 (51.6–67.9)
Right	56 (49–64)	58.5 (49.7–65.0)	NR	NA	63.8 (55.0–71.5)	59.8 ± 12.2	63.9 (61.2–66.7)	70 (63.5–77.5)	59.3 (51.3–67.9)
Left	58 (50–66)	57.0 (49.7–64.0)	NR	NA	59.1 (49.7–66.6)	57.6 ± 10.3	61 (59–63)	67 (58–74)	60.3 (52.0–68.0)
**Gender (females %)**	41.8%	38.8%	46.2%	NA	36%	34.4%	35.2%	41.4%	42.9%
Right	44.5%	53%	NR	NA	43.8%	43.9%	44%	43.1%	49%
Left	40.4%	35%	NR	NA	33%	32.3%	33%	43.2%	35%
**Presentation of liver metastasis (synchronous %)**	74.1%	51.2%	100%	64.4%	48.2%	100%	63.2%	62.4%	53.4%
Right	76%	52.9%	100%	63.9%	46.4%	100%	64.1%	63.2%	55%
Left	73.1%	50.8%	100%	62.6%	49%	100%	62.1%	70.8%	54.8%
**Number of liver metastases Median [IQR] OR Multiple %**	Reported but on different ^a^	NR	NR	2 (1–4)	2 (1–3)	Reported but on different ^b^	Reported but on different ^c^	3 (1–4)	2 (1–3)
Right		2 (1–3.5)	NR	2 (1–3)	2 (1–3)	NR	NR	3 (1–4)	2 (1–3)
Left		2 (1–4)	NR	2 (1–4)	2 (1–4)	NR	NR	3 (1–4)	2 (1–3)
**Concurrent extrahepatic disease**	NR	11.9%	NR	8.7%	10.8%	NR	0%	18.3%	7.19
Right		12.7%	NR	9.5%	11.8%	NR	0%	18%	5.7%
Left		11.6%	NR	8.2%	10.5%	NR	0%	21.1%	8.2%
**R0 resections (*n*, %)**	92.8%	NR	85.7%	89.4%	80.2%	79.7%	98.1%	98.7%	95.2%
Right	91.6%	NR	NR	89.6%	79.8%	85.4%	97.3%	98.9%	95.3%
Left	93.4%	NR	NR	91.5%	81.3%	78.5%	99.3%	98.1%	95.2%
**Modern prehepatectomy chemotherapy**	99%	NR	NR		65%	NR	23%	41%	91.9%
Right	99%	NR	NR	NR	59%	NR	20%	43.4%	91.9%
Left	100%	NR	NR	NR	67.5%	NR	24%	40.2%	91.6%
**Biologic agents**	55%	NR	NR	NR	30.7%	42.3%	10.2%	32.6%	29.3%
Right	55%	NR	NR	NR	28%	31.7%	8%	35.4%	24%
Left	55%	NR	NR	NR	31.7%	44.6%	13%	32%	37%
**Major response to chemotherapy** ^†^	68%	NR	NR	NR	NR	NR	NR	NR	NR
Right	62%	NR	NR	NR	NR	NR	NR	NR	NR
Left	71%	NR	NR	NR	NR	NR	NR	NR	NR
**Adjuvant chemotherapy**	66.2%	56.7%	91%	93.2%	58.3%	91.6%	85%	30.2%	96.5%
Right	65.9%	45.3%	NR	92.3%	56.5%	85.4%	82.3%	28.6%	93.6%
Left	66.3%	59.6%	NR	93.6%	62.8%	93%	88.8%	31%	97.1%

^a^ The study reported tumor number as solitary vs. multiple. ^b^ The study reported tumor number as <3 vs. ≥3. ^c^ The study reported tumor number as ≤3 vs. >3 * University of Lyon and Clermont-Ferrand University.; ^†^ biologic agents: anti-VEGF or anti-EGFR; modern chemotherapy: oxaliplatin- or irinotecan-based regimens; major response to chemotherapy: viable tumor 0–49%.

## Data Availability

The data sharing of individual patient data from each participating center will be subject to the policy and procedures of the institutions and groups who conducted the original study.

## Supplementary Materials

The following supporting information can be downloaded at: https://www.mdpi.com/article/10.3390/cancers14030799/s1. eFile S1: PRISMA 2020 item checklist, eFile S2: Search terms.

## References

[1] Loree J., Pereira A., Lam M., Willauer A., Raghav K., Dasari A., Morris V.K., Advani S.M., Menter D.G., Eng C. (2018). Classifying Colorectal Cancer by Tumor Location Rather than Sidedness Highlights a Continuum in Mutation Profiles and Consensus Molecular Subtypes. Clin. Cancer Res..

[2] Arnold D., Lueza B., Douillard J.-Y., Peeters M., Lenz H.-J., Venook A., Heinemann V., Van Cutsem E., Pignon J.-P., Tabernero J. (2017). Prognostic and predictive value of primary tumour side in patients with RAS wild-type metastatic colorectal cancer treated with chemotherapy and EGFR directed antibodies in six randomized trials. Ann. Oncol..

[3] Cremolini C., Antoniotti C., Lonardi S., Bergamo F., Cortesi E., Tomasello G., Moretto R., Ronzoni M., Racca P., Loupakis F. (2018). Primary tumor sidedness and benefit from FOLFOXIRI plus bevacizumab as initial therapy for metastatic colorectal cancer. Retrospective analysis of the TRIBE trial by GONO. Ann. Oncol..

[4] Sasaki K., Andreatos N., Margonis G.A., He J., Weiss M., Johnston F., Wolfgang C., Antoniou E., Pikoulis E., Pawlik T.M. (2016). The prognostic implications of primary colorectal tumor location on recurrence and overall survival in patients undergoing resection for colorectal liver metastasis. J. Surg. Oncol..

[5] Creasy J.M., Sadot E., Koerkamp B.G., Chou J.F., Gonen M., Kemeny N.E., Saltz L.B., Balachandran V.P., Kingham T.P., DeMatteo R.P. (2017). The Impact of Primary Tumor Location on Long-Term Survival in Patients Undergoing Hepatic Resection for Metastatic Colon Cancer. Ann. Surg. Oncol..

[6] Scherman P., Syk I., Holmberg E., Naredi P., Rizell M. (2020). Influence of primary tumour and patient factors on survival in patients undergoing curative resection and treatment for liver metastases from colorectal cancer. BJS Open.

[7] Wang K., Xu D., Yan X.-L., Poston G., Xing B.-C. (2018). The impact of primary tumour location in patients undergoing hepatic resection for colorectal liver metastasis. Eur. J. Surg. Oncol. (EJSO).

[8] Wang X.-Y., Zhang R., Wang Z., Geng Y., Lin J., Ma K., Zuo J.-L., Lu L., Zhang J.-B., Zhu W.-W. (2019). Meta-analysis of the association between primary tumour location and prognosis after surgical resection of colorectal liver metastases. Br. J. Surg..

[9] Margonis G.A., Amini N., Buettner S., Kim Y., Wang J., Andreatos N., Wagner D., Sasaki K., Beer A., Kamphues C. (2021). The Prognostic Impact of Primary Tumor Site Differs According to the KRAS Mutational Status: A Study By the International Genetic Consortium for Colorectal Liver Metastasis. Ann. Surg..

[10] Yamashita S., Brudvik K.W., Kopetz S., Maru D., Clarke C.N., Passot G., Conrad C., Chun Y.S., Aloia T.A., Vauthey J.-N. (2018). Embryonic Origin of Primary Colon Cancer Predicts Pathologic Response and Survival in Patients Undergoing Resection for Colon Cancer Liver Metastases. Ann. Surg..

[11] Page M.J., McKenzie J.E., Bossuyt P.M., Boutron I., Hoffmann T.C., Mulrow C.D., Shamseer L., Tetzlaff J.M., Akl E.A., Brennan S.E. (2021). The PRISMA 2020 statement: An updated guideline for reporting systematic reviews. BMJ.

[12] Higgins J.P., Thomas J., Chandler J., Cumpston M., Li T., Page M.J., Welch V.A. (2019). Cochrane Handbook for Systematic Reviews of Interventions.

[13] Guyot P., Ades A.E., Ouwens M.J.N.M., Welton N.J. (2012). Enhanced secondary analysis of survival data: Reconstructing the data from published Kaplan-Meier survival curves. BMC Med. Res. Methodol..

[14] Seide S.E., Röver C., Friede T. (2019). Likelihood-based random-effects meta-analysis with few studies: Empirical and simulation studies. BMC Med. Res. Methodol..

[15] IntHout J., Ioannidis J.P., Borm G.F. (2014). The Hartung-Knapp-Sidik-Jonkman method for random effects meta-analysis is straightforward and considerably outperforms the standard DerSimonian-Laird method. BMC Med. Res. Methodol..

[16] IntHout J., Ioannidis J.P., Rovers M.M., Goeman J.J. (2016). Plea for routinely presenting prediction intervals in meta-analysis. BMJ Open.

[17] Sterne J.A.C., Sutton A.J., Ioannidis J.P.A., Terrin N., Jones D.R., Lau J., Carpenter J., Rücker G., Harbord R.M., Schmid C.H. (2011). Recommendations for examining and interpreting funnel plot asymmetry in meta-analyses of randomised controlled trials. BMJ.

[18] Goffredo P., Utria A.F., Beck A.C., Chun Y.S., Howe J., Weigel R.J., Vauthey J.-N., Hassan I. (2018). The Prognostic Impact of KRAS Mutation in Patients Having Curative Resection of Synchronous Colorectal Liver Metastases. J. Gastrointest. Surg..

[19] Gagnière J., Dupré A., Gholami S.S., Pezet D., Boerner T., Gönen M., Kingham T.P., Allen P.J., Balachandran V.P., De Matteo R.P. (2020). Is Hepatectomy Justified for BRAF Mutant Colorectal Liver Metastases? A Multi-institutional Analysis of 1497 Patients. Ann. Surg..

[20] Kim H.S., Lee J.M., Kim H.S., Yang S.Y., Han Y.D., Cho M.S., Hur H., Min B.S., Lee K.Y., Kim N.K. (2020). Prognosis of Synchronous Colorectal Liver Metastases After Simultaneous Curative-Intent Surgery According to Primary Tumor Location and KRAS Mutational Status. Ann. Surg. Oncol..

[21] Chen T.-H., Chen W.-S., Jiang J.-K., Yang S.-H., Wang H.-S., Chang S.-C., Lan Y.-T., Lin C.-C., Lin H.-H., Huang S.-C. (2021). Effect of Primary Tumor Location on Postmetastasectomy Survival in Patients with Colorectal Cancer Liver Metastasis. J. Gastrointest. Surg..

[22] O’Connor J.M., Sanchez Loria F., Ardiles V., Grondona J., Sanchez P., Andriani O., Fauda M., Brancato F., Huertas E., Alvarez F. (2019). Prognostic impact of K-RAS mutational status and primary tumor location in patients undergoing resection for colorectal cancer liver metastases: An update. Future Oncol..

[23] Kamphues C., Kadowaki S., Amini N., Berg I.V.D., Wang J., Andreatos N., Sakamoto Y., Ogura T., Kakuta M., Pikouli A. (2021). The interplay of KRAS mutational status with tumor laterality in non-metastatic colorectal cancer: An international, multi-institutional study in patients with known KRAS, BRAF, and MSI status. J. Surg. Oncol..

[24] Brudvik K.W., Kopetz S., Li L., Conrad C., Aloia T.A., Vauthey J. (2015). Meta-analysis of KRAS mutations and survival after resection of colorectal liver metastases. Br. J. Surg..

[25] Yin J., Cohen R., Jin Z., Liu H., Pederson L., Adams R., Grothey A., Maughan T.S., Venook A., Van Cutsem E. (2021). Prognostic and Predictive Impact of Primary Tumor Sidedness for Previously Untreated Advanced Colorectal Cancer. JNCI J. Natl. Cancer Inst..

[26] Cavallaro P., Bordeianou L., Stafford C., Clark J., Berger D., Cusack J., Kunitake H., Francone T., Ricciardi R. (2019). Impact of Single-organ Metastasis to the Liver or Lung and Genetic Mutation Status on Prognosis in Stage IV Colorectal Cancer. Clin. Color. Cancer.

[27] Kawaguchi Y., Kopetz S., Newhook T.E., De Bellis M., Chun Y.S., Tzeng C.-W.D., Aloia T.A., Vauthey J.-N. (2019). Mutation Status of RAS, TP53, and SMAD4 is Superior to Mutation Status of RAS Alone for Predicting Prognosis after Resection of Colorectal Liver Metastases. Clin. Cancer Res..

[28] Datta J., Smith J.J., Chatila W.K., McAuliffe J.C., Kandoth C., Vakiani E., Frankel T.L., Ganesh K., Wasserman I., Lipsyc-Sharf M. (2020). Coaltered Ras/B-raf and TP53 Is Associated with Extremes of Survivorship and Distinct Patterns of Metastasis in Patients with Metastatic Colorectal Cancer. Clin. Cancer Res..

[29] Nordlinger B., Sorbye H., Glimelius B., Poston G.J., Schlag P.M., Rougier P., Bechstein W.O., Primrose J.N., Walpole E.T., Finch-Jones M. (2008). Perioperative chemotherapy with FOLFOX4 and surgery versus surgery alone for resectable liver metastases from colorectal cancer (EORTC Intergroup trial 40983): A randomised controlled trial. Lancet.

[30] Santini D., Loupakis F., Vincenzi B., Floriani I., Stasi I., Canestrari E., Rulli E., Maltese P.E., Andreoni F., Masi G. (2008). High Concordance of KRAS Status Between Primary Colorectal Tumors and Related Metastatic Sites: Implications for Clinical Practice. Oncologist.

[31] Margonis G.A., Andreatos N., Kreis M.E., D’Angelica M. (2020). The Interplay of Primary Tumor Location and KRAS Mutation Status in Patients with Synchronous Colorectal Cancer Liver Metastases: Current Data and Unanswered Questions. Ann. Surg. Oncol..

[32] Amram M., Dunn J., Zhuo Y.D. (2020). Optimal Policy Trees. arXiv.

