# Supplementary material for: Is Laterality Prognostic in Resected KRAS-Mutated Colorectal Liver Metastases? A Systematic Review and Meta-Analysis
Source: Cancers (Basel). 2022 Feb 4;14(3):799. doi: 10.3390/cancers14030799 (PMC8833748; doi:10.3390/cancers14030799)
Supplement: Supplementary file 1 [file cancers-14-00799-s001.zip › eFile S2.pdf]

**eFile S2. Search terms**

((((((("colorectal neoplasms"[MeSH Terms] “Colon cancer”[Tiab] OR “Colorectal Neoplasms/mortality”[MeSH Terms] OR ((colon[mesh] OR colon[tiab] OR colonic[tiab] OR colorectal[tiab] OR rectal[tiab]) AND (cancer[Tiab] OR cancers[Tiab] OR carcinoma[Tiab] OR carcinomas[Tiab] OR adenocarcinoma[Tiab] OR adenocarcinomas[Tiab] OR tumour[Tiab] OR "neoplasms"[MeSH Terms] OR neoplasm[Tiab] OR neoplasms[Tiab] OR tumours[Tiab] OR tumor[Tiab] OR tumors[Tiab])))))) AND (((right[Tiab] OR left[Tiab] OR site[Tiab] OR sites [tiab] OR side[Tiab] OR sidedness[Tiab] OR sideness[Tiab] OR "functional laterality"[MeSH Terms] OR "laterality"[Tiab] OR descending[Tiab] OR sigmoid[Tiab] OR proximal[Tiab] OR distal[Tiab] OR caecum[Tiab] OR cecum[Tiab] OR “primary location”[tiab] OR primary tumor location[tiab] OR primary tumor site[tiab] OR primary tumour location[tiab] OR "colon, sigmoid"[MeSH Terms] OR "cecum"[MeSH Terms] OR ”primary tumor location”[tiab]))))  AND (((surgery[subheading] OR resect*[Tiab] OR surgical[Tiab] OR surgery[Tiab] OR operation*[tiab] OR operate*[tiab] OR "Surgical Procedures, Operative"[Mesh] OR hepatect*[Tiab] OR hepatectomy[tiab])))) AND (((Liver neoplasms[MeSH Terms] OR Neoplasm Metastasis[MeSH Terms] OR liver*[tw] OR hepati*[tw] OR hepato*[tw] OR Hepatectomy/mortality*[MeSH Terms])))) AND ((("ras Proteins"[MeSH Terms] OR K-RAS[tiab] OR “ KRAS Mutational Status”[tiab] OR “ BRAF Mutational Status”[tiab]OR KRAS protein[MeSH Terms] OR mutation[tiab] OR mutational[tiab] OR mutated[tiab] OR mutations[tiab] OR ras[tiab] OR kras[tiab] OR k-ras[tiab] OR ki-ras[tiab] OR codon 12[tiab] OR codon 13[tiab] OR exon 2[tiab] OR BRAF[tiab] OR “BRAF Mutant”[tiab] OR Prognostic[tiab] OR “prognostic”[tiab] OR “prognostic impact”[tiab] OR “Colorectal Liver Metastases”[tiab] )))
